# A Rare Case of Bronchomediastinal Pulmonary Vein Fistula due to Fibrosing Mediastinitis

**DOI:** 10.7759/cureus.10439

**Published:** 2020-09-14

**Authors:** Harsh Rawal, Sugandhi Mahajan, Andrea Brasch, Vishesh Paul

**Affiliations:** 1 Internal Medicine, University of Connecticut, Hartford, USA; 2 Internal Medicine, Carle Foundation Hospital, Urbana, USA; 3 Cardiology, Carle Foundation Hospital, Urbana, USA; 4 Pulmonology and Critical Care Medicine, Carle Foundation Hospital, Urbana, USA

**Keywords:** fibrosing mediastinitis, histoplasmosis, bronchomediastinal pulmonary vein fistula, transesophageal echocardiogram

## Abstract

Fibrosing mediastinitis (FM) is a rare condition with extensive proliferation of fibrous tissue in the mediastinum usually happens few years after Histoplasma infection. FM usually occurs years later after presentation of Histoplasma infection, and usually what makes patients seek medical attention are symptoms from compression and occlusion of vital mediastinal structures, such as the central airways, superior vena cava, pulmonary arteries, and veins. Rarely, heart, pericardium, coronaries, and aorta are involved. We report a case of 39-year-old-male who was admitted with fever and cough. The patient’s condition worsened despite being on broad-spectrum antibiotics, with worsening encephalopathy and a new onset lower extremity weakness. Brain imaging showed multiple strokes suggestive of embolic event. CT chest/abdomen was suggestive of FM along with cavitary lung nodules and pneumomediastinum. Splenic and renal infarcts were also noted. Infective endocarditis was one of the top differential diagnosis due to multiple embolic infarcts, and hence a transesophageal echocardiography (TEE) was pursued. TEE showed a mass along with air bubbles entering the left atrium from the pulmonary vein. On re-evaluation of CT chest images, a fistula was seen extending from the mediastinum to the left main bronchus and the left upper pulmonary vein. This supported the diagnosis of FM with erosion of lymph node into the left main bronchus and left upper pulmonary artery, leading to fistula formation and subsequent systemic air embolization. The diagnosis of FM requires a multimodality approach, high clinical suspicion, and accurate history taking. Treatment mainly aims at managing the mechanical complications.

## Introduction

Fibrosing mediastinitis (FM) is a rare condition characterized by fibroinflammatory changes in the mediastinum. These changes are usually associated with proliferation of acellular collagen and fibrous tissue. Many cases are idiopathic; however, certain infectious organisms like Histoplasma do play a role by eliciting an immune response. Most of the patients were historically noted to be young individuals. This presentation has been noted to occur few years after Histoplasma infection. There are two types of FM that have been described: focal and diffuse. The focal type is usually localized to subcarinal and paratracheal areas, whereas the diffuse type affects multiple mediastinal compartments. The clinical presentation could vary from incidental imaging finding to diverse symptoms from compression and occlusion of various mediastinal structures. Imaging modalities with CT scan and MRI are the usual modes of diagnosis [1]. We report a rare case of FM complicated by bronchomediastinal pulmonary vein fistula causing catastrophic septic emboli and further systemic sequelae.

## Case presentation

A 39-year-old male was admitted to an outside hospital with persistent fevers, chills, and cough. Past medical history was significant for pulmonary histoplasmosis that was treated two years ago. Chest imaging at that time showed FM with lymphadenopathy without any complications. His current symptoms were present for two weeks, and he failed the outpatient treatment for community-acquired pneumonia. Chest X-ray showed enlarged mediastinal lymph nodes with a cavitary lung nodule. The patient was started on broad-spectrum antibiotics. On second day of hospitalization, the patient developed acute encephalopathy and left lower extremity weakness. MRI brain showed multiple areas of diffusion restriction suggestive of embolic phenomena. Due to worsening condition, the patient was transferred to our facility.

On presentation, vitals were as follows: temperature 104˚F, heart rate (HR) 130/min, respiratory rate (RR) 24/min, and blood pressure (BP) 150/75 mmHg. Heart sounds were normal, and there were scattered crackles on auscultation in both lungs. Neurologically, he had weakness of left lower extremity. Blood work was significant for leukocytosis of 24 × 10^9^/L. Blood culture and fungal workup were sent. The patient was started on antibiotics and antifungals.

**Figure 1:**
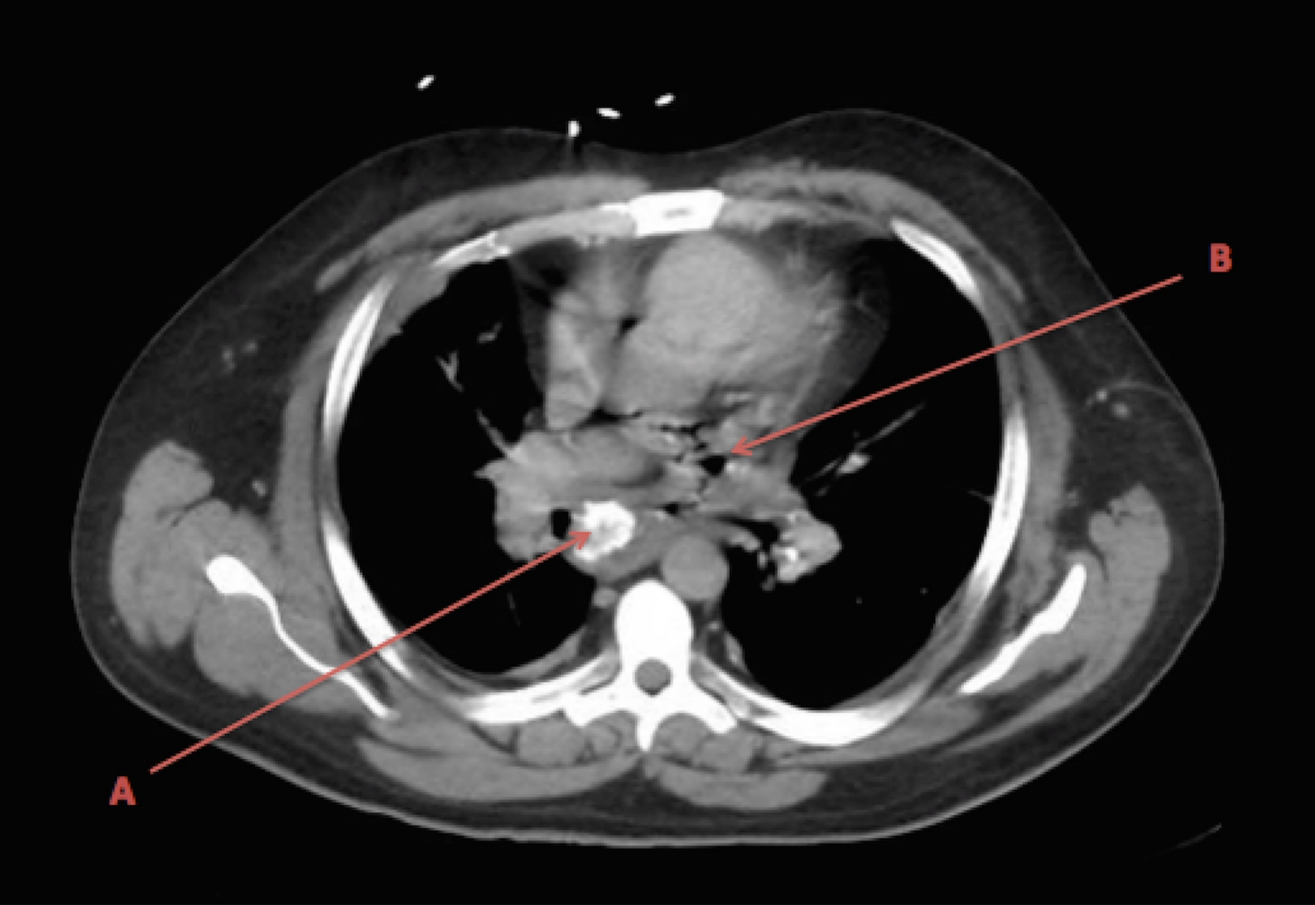
CT demonstrating calcified lymphnode (A) and pneumomediastinum (B) due to the fistula

CT chest/abdomen demonstrated cavitary lung nodules, mediastinal lymphadenopathy, and pneumomediastinum (Figure 1).

**Figure 2:**
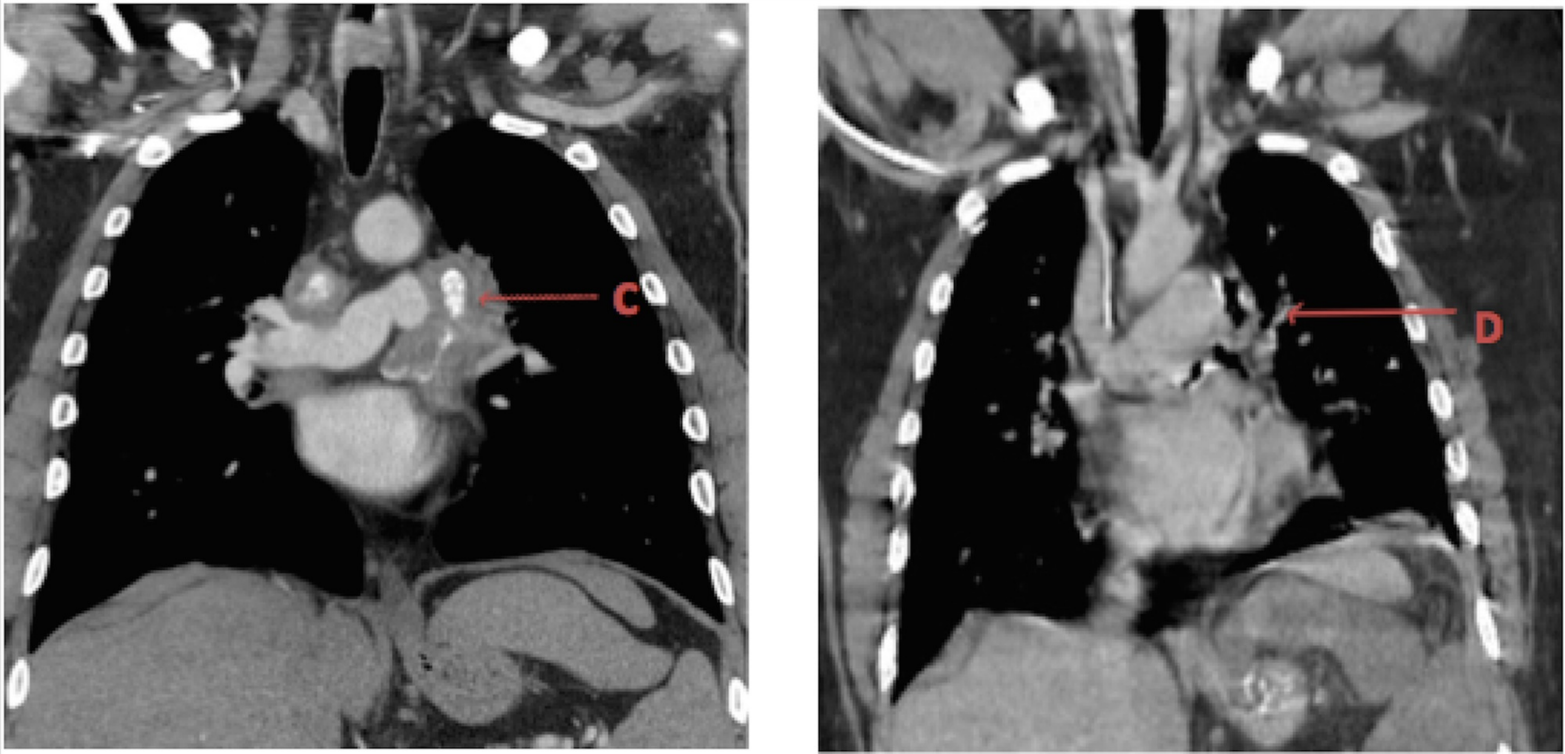
CT images comparing previous (left) and current (right) imaging with lymph node eroding into the left bronchus and upper pulmonary vein with new pneumomediastinum. C: Fibrosing mediastinitis seen in the CT chest two years ago. D: Erosion of lymph node into the left bronchus and the left upper pulmonary vein

**Figure 3:**
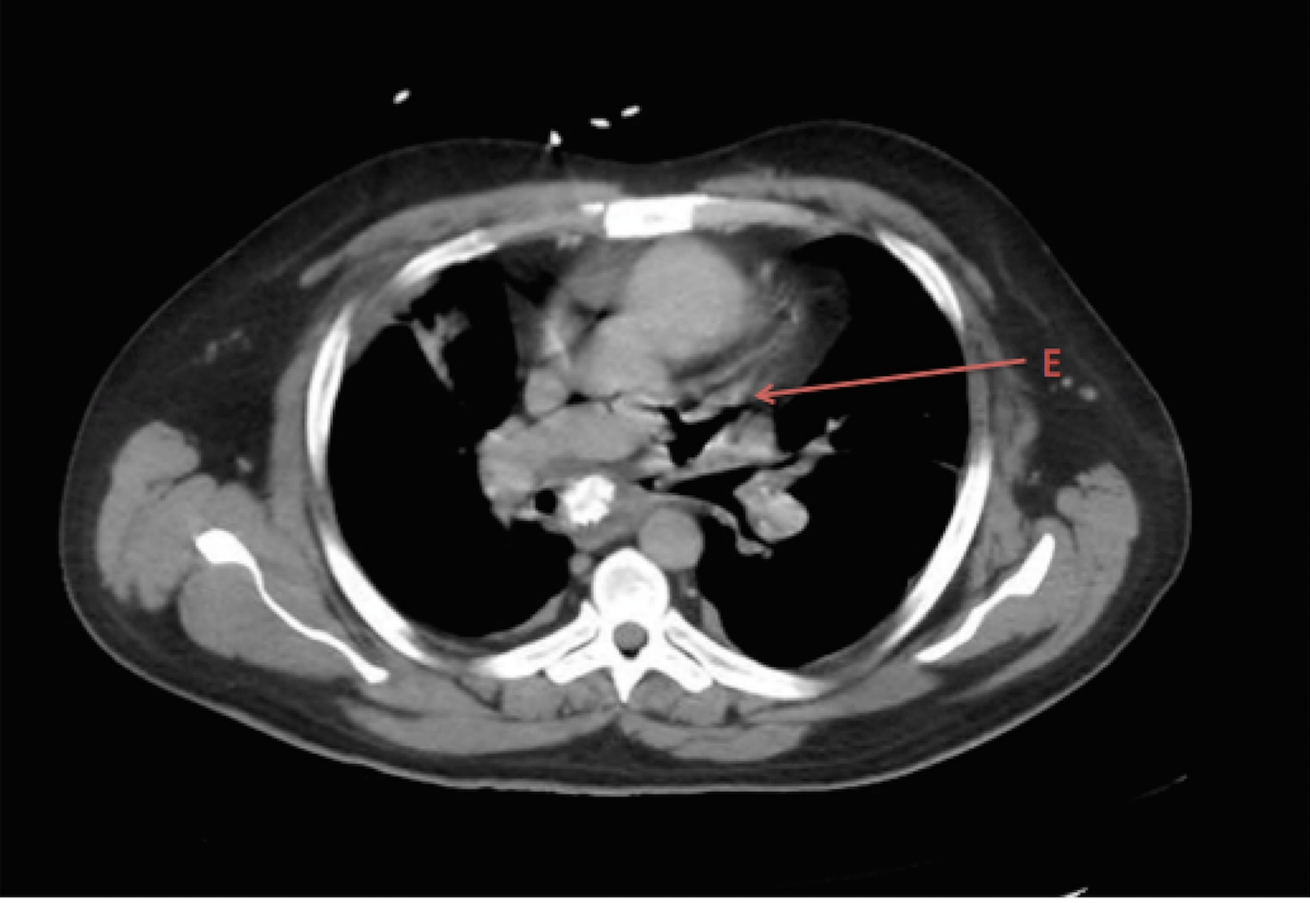
E: Axial plane CT scan showing fistulous tract between mediastinum, left upper pulmonary vein, and left mainstem bronchus

Splenic and renal infarcts were also noted. Imaging findings from two years ago were reviewed and compared. The patient had FM from before but the findings of pneumomediastinum were new (Figures 2, 3).

**Figure 4:**
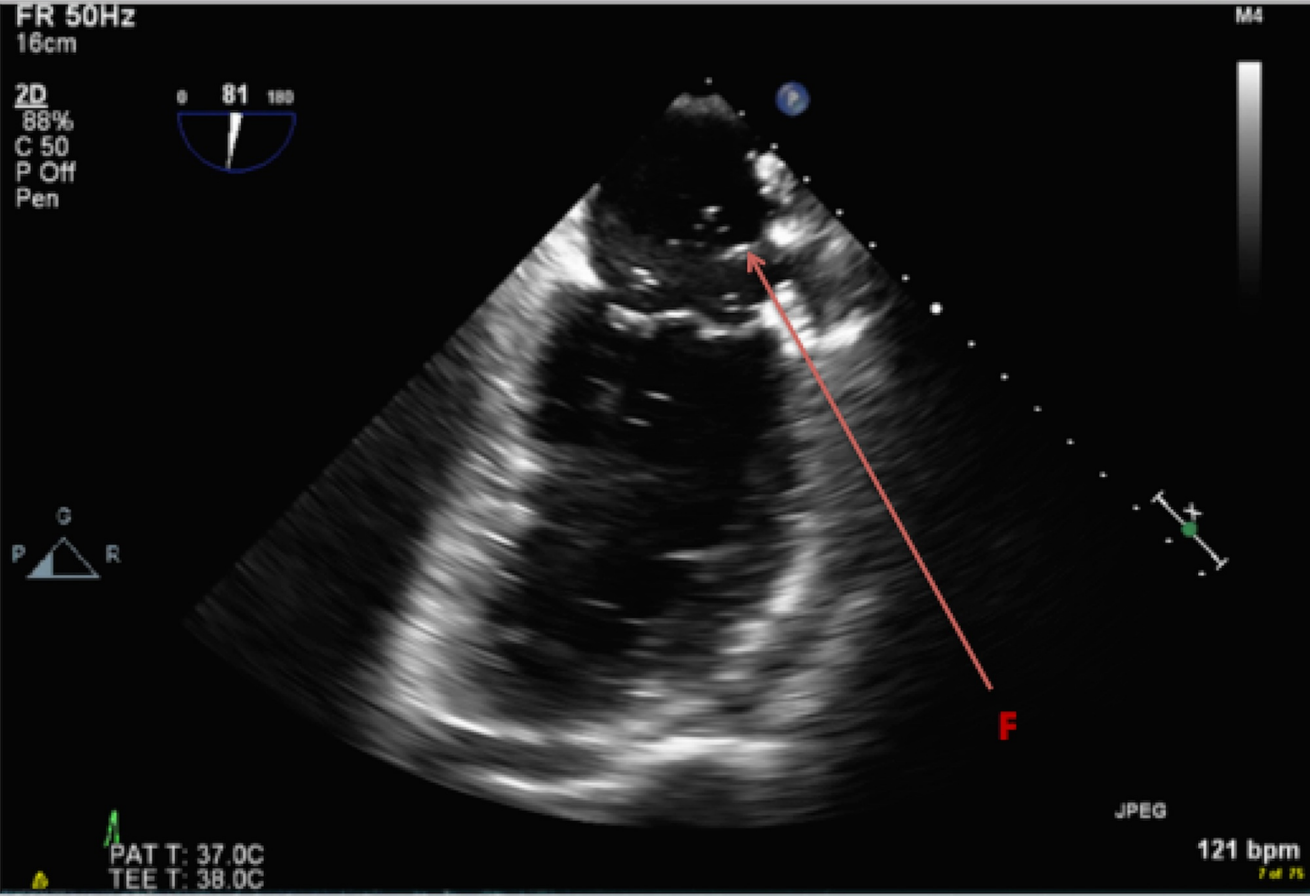
F: Transesophageal echocardiogram (TEE) demonstrating mediastinal mass extending into the left atrium from the left upper pulmonary vein

Transesophageal echocardiography (TEE) was pursued to investigate for source of systemic embolization. No vegetations or thrombus were seen, but a mass was observed protruding from left upper pulmonary vein along with air bubbles streaming into left atrium (Figure 4).

Taking together the findings of FM, pneumomediastinum, and mass in pulmonary vein along with air bubbles, the CT chest images were reviewed again with the radiology team. The possibility of fistulous track extending between mediastinum, left mainstem bronchus, and left pulmonary vein was suggested as per the imaging above. The fistula was allowing air to continuously move from bronchus to left side of heart causing air embolization systemically and to mediastinum leading to pneumomediastinum. This supported the diagnosis of FM with erosion of lymph node into the left main bronchus and the left upper pulmonary vein, leading to fistula formation. The patient was referred for surgical exploration, but unfortunately his condition worsened and he succumbed to his illness due to cardiac arrest.

## Discussion

FM is a rare condition characterized by extensive proliferation of fibrous tissue in the mediastinum. While actual data are not available, it has been estimated that of the 500,000 people infected with Histoplasma every year, less than 1% developed FM [2]. FM can be idiopathic or secondary to an etiological agent. Separate data on the incidence and prevalence of each type are limited since the number of cases is not very large. It most commonly occurs as a late consequence of Histoplasma infection. Other causes include tuberculosis, radiation, malignancy, sarcoidosis, and autoimmune diseases [3]. Radiographically, two distinct patterns of involvement have been described. The focal pattern is most likely due to histoplasmosis and manifest as calcified mass in the subcarinal, paratracheal, and hilar regions. On the other hand, the diffuse pattern is usually idiopathic and manifests as diffusely infiltrating non-calcified mass that involves multiple mediastinal compartments [4].

The symptoms depend on site of involvement leading to compression, obstruction, and invasion of various mediastinal structures. The involvement of right paratracheal region can lead to superior vena cava and azygous vein compression. The involvement of subcarinal area can cause obstruction of bronchi, and erosions of the calcified mass into the bronchus can lead to broncholithiasis. The lateral extension of fibrosing process in subcarinal region can lead to compression of pulmonary artery, while the anterior extension can compromise the pulmonary venous flow. Posterior mediastinal extension leads to obstruction of esophagus. Rarely, heart, pericardium, coronaries, and aorta can also be involved [5].

Chest X-rays can reveal calcified mediastinal and hilar lymphadenopathy. Contrast-enhanced CT chest can show variable enhancement for fibrous tissue and help to delineate the extent of visceral involvement. Echocardiogram helps to identify cardiac involvement, and lung perfusion scan can show the compromised pulmonary blood flow. The diagnosis is usually made based on clinical and radiographic presentation, and tissue sampling is rarely required [6]. No pharmacological therapy has been shown to affect the outcome, but some clinicians have used antifungals in post-histoplasmosis FM with some success. In our case, the patient presented with complications from compressive symptoms. He was treated with antifungal therapy two years ago prior to presentation. Steroids have also shown radiographic evidence of improvement in some non-infectious cases [4]. Treatment mainly involves managing the mechanical problems, and it may also involve intravascular and airway stents and pneumonectomy [7].

Despite advances in medicine, FM remains a mystery, without definitive cure. FM is usually a non-progressive disease but mechanical complications can lead to catastrophic outcomes as was seen in our patient [8]. Bronchomediastinal pulmonary vein fistula is an extremely rare complication. It has been hard to quantify the prevalence of this complication since the incidence of FM itself is less than 1% of the patients who had Histoplasma infection. Amongst the multitude of compressive symptoms that patients can present with, a fistula with three different structures was not frequently found. Imaging findings of pneumomediastinum and systemic air embolization in our patient lead to the final diagnosis of FM complicated with fistula formation.

## Conclusions

This case highlights the importance of broad differential diagnosis when dealing with diseases causing multisystem organ failure. Patients with past history of Histoplasma infection should prompt the differential diagnosis of FM. Younger population is at a risk as well. Compressive symptoms can lead to life-threatening complications, and hence high suspicion is necessary. Fistula amongst various mediastinal structures is a rare manifestation of FM, and early diagnosis and management can help avert the catastrophic outcomes.
